# Commentary on: A Comprehensive Mechanical and Chemoprophylaxis Algorithm for Prevention of Venous Thromboembolism in Lipoabdominoplasty

**DOI:** 10.1093/asjof/ojaf118

**Published:** 2025-09-20

**Authors:** Christopher J Pannucci

See the Original Article here.

I appreciate the opportunity to write an Invited Commentary for Claytor et al's manuscript titled “A Comprehensive Mechanical and Chemoprophylaxis Algorithm for Prevention of Venous Thromboembolism in Lipoabdominoplasty.”^1^ Dr Claytor and I met at Washington University in St. Louis 22 years ago, when I was a medical student and he was a hand surgery fellow. Since at least 2014, we have had a running conversation about venous thromboembolism (VTE), from the podium and the microphone at meetings, over emails and phone calls, and while sitting on panels together. We have differing opinions, as you will see, and I hope that an alternate perspective helps the reader to better clarify their own interpretation of the data and support their own personal practices.

## THOUGHTFUL CONSIDERATION OF VENOUS THROMBOEMBOLISM RISK

The manuscript title suggests that Claytor et al's “comprehensive” approach includes both mechanical prophylaxis and prophylactic dose anticoagulation. However, the described process of peri-operative optimization goes far beyond these 2 factors, to incorporate existing paradigms of risk identification, risk modification, and risk reduction.^2^ Specifically, the 2005 Caprini score was used to identify baseline VTE risk level. Patient-centric risk was modified actively and pre-emptively, through estrogen cessation, smoking cessation, and hematology consultation in higher-risk patients. Risk reduction during surgery was performed through universal use of sequential compression devices, and the role of plication in intra-abdominal pressure elevation was acknowledged. Early ambulation occurred amongst this outpatient population. This multi-faceted and proactive approach to VTE risk is in line with current published paradigms of care.^3^

## THE ROLE OF HEMATOLOGY CONSULTATION

A thoughtful process, such as the one described by the authors, identifies multiple opportunities for patients or surgeons to take the “off ramp”: presumably, there were patients seen in consultation who were not offered surgery based on the surgeon's perception of risk, or who declined surgery based on the discussed risk level. Patients with a personal history of VTE, or a first-degree relative with VTE, were sent for hematology consultation. Claytor et al note that, among 25 patients (7.5% of the cohort) sent for hematology consultation, 100% were cleared for surgery. Similar to Claytor et al's experience, I am yet to have a hematologist tell an elective patient that surgery risks outweigh surgery benefits. For elective procedures, I often find my elective surgery criteria to be more stringent than the consulting hematologist's. This may be a reflection of my own level of risk tolerance, but elective procedures cannot move forward if the patient or the surgeon is not comfortable. My personal goals in hematology consultation, when requested, are to ensure a hypercoagulability workup is completed correctly, and to afford patients the opportunity for additional conversation surrounding baseline VTE risk as well as VTE risk modification and reduction. My patients understand that the hematologist does not make the yes/no decision for surgery. For elective surgery, I have patients return to the clinic after hematology consultation, so that we can discuss the risks and benefits of surgery, moving forward only if we are aligned in our risk/benefit tolerances.

## THE MAJORITY OF VENOUS THROMBOEMBOLISM AFTER ABDOMINOPLASTY OCCURS IN LOWER-RISK PATIENTS

The majority of VTE events after abdominoplasty occur in patients with lower Caprini scores, and the authors note that “a vast majority of patients are stratified as low/moderate risk and yet paradoxically, this cohort has a higher numerical incidence of PE than high risk patients.”^4^ Some have used these data, originally published by Keyes out of the AAASF database, to question the utility of Caprini scores in the aesthetic population.^4^ My Stanford University colleagues and I have previously used simulation studies to show why most VTE events occur in lower-risk patients. Specifically, using 1 million simulations in a Monte Carlo model, we demonstrated that a rare event in a large population occurs more frequently than a more common event in a very small population.^5^ Based on those modeling studies, the expectation is that the majority of VTE events after abdominoplasty should occur in patients with lower 2005 Caprini scores. This is because of probability and proportions, not a problem with the Caprini score.

Claytor et al's own introduction provides clarity on the composition of the abdominoplasty population, suggesting that “a reasonable strategy is to avoid operating on ‘high risk’ patients and therefore, approximately 97% of patients are stratified as low/moderate risk.” Claytor et al's own data confirms this-only 4.8% of the described abdominoplasty population had Caprini ≥7, the level at which anticoagulation is known to have a favorable risk/benefit for plastic surgery inpatients.^6^ Claytor et al are not unique in operating on a generally heavily selected, low risk population. Data from others have shown that a minority, specifically 1% of tummy tuck, 2% of the rhinoplasty population, and 4% of breast augmentation patients, have Caprini ≥7.^5,7,8^ My interpretation of these data are reflected in the title of a 2016 Invited Commentary for the *Aesthetic Surgery Journal*: “The vast majority of aesthetic surgery patients are at low risk of VTE and do not require chemical prophylaxis.”^9^ This title, published nearly 10 years ago, reflects where Claytor et al's interpretation and others (including mine) differ.

## UNIVERSAL ANTICOAGULATION AFTER ABDOMINOPLASTY

Is there a harm in universal anticoagulation? Claytor et al justify the intervention thusly:

This study suggests implementation of a far more aggressive chemo- and mechanical prophylaxis protocols than the current accepted regimens is a safe and effective measure in preventing VTE. In doing so, surgeons may be able to provide widespread protection against mortality secondary to PE while having minimal, if any, impact on hematoma incidence.

Claytor et al's goal is to prevent mortality, and they provide supporting data by considering the 2 non-fatal PE in the series. But, the authors correctly point out that “Perhaps a less aggressive protocol can prevent VTE just as well as the proposed method.” The best data assessing the prophylactic dose anticoagulation's impact remains the VTEPS study. That multicenter, Plastic Surgery Foundation study examined VTE rates among Caprini-stratified inpatients who did or did not receive enoxaparin 40 mg once daily for the duration of inpatient stay. The study showed, via multivariable regression, that only Caprini 7 to 8 and >8 inpatients who received enoxaparin had a significant 60-day VTE risk reduction.^6^ This relationship was confirmed in a larger meta-analysis involving over 14,000 surgery patients from 11 clinical trials.^10^ Broad anticoagulation comes at a cost; that same meta-analysis confirmed significant elevation in population-level bleeding when surgical patients received prophylactic dose anticoagulation. Claytor et al's study population had a 1.8% rate of post-operative bleeding, in line with other studies providing universal anticoagulation after abdominoplasty.^11-13^

## DECISIONS ON ANTICOAGULATION: NOW AND IN THE FUTURE

In abdominoplasty, Claytor et al support universal anticoagulation.^11-13^ I advocate for anticoagulation guided by the 2005 Caprini score and surgery factors. Others advocate for no anticoagulation in any patient.^14^ The correct answer probably lies somewhere between these positions. For the present, surgeons must review currently available data, and with their patients, make the best decisions based on clinical circumstances and risk tolerance. For the future, I look forward to ongoing research by Claytor et al and others to provide increased clarity on this important patient safety issue.
